# Cell-type specific transcriptomic signatures of neocortical circuit organization and their relevance to autism

**DOI:** 10.3389/fncir.2022.982721

**Published:** 2022-09-23

**Authors:** Anthony J. Moussa, Jason C. Wester

**Affiliations:** Department of Neuroscience, The Ohio State University College of Medicine, Columbus, OH, United States

**Keywords:** neocortex, circuits, neuronal class, transcriptomics, regionalization

## Abstract

A prevailing challenge in neuroscience is understanding how diverse neuronal cell types select their synaptic partners to form circuits. In the neocortex, major classes of excitatory projection neurons and inhibitory interneurons are conserved across functionally distinct regions. There is evidence these classes form canonical circuit motifs that depend primarily on their identity; however, regional cues likely also influence their choice of synaptic partners. We mined the Allen Institute’s single-cell RNA-sequencing database of mouse cortical neurons to study the expression of genes necessary for synaptic connectivity and physiology in two regions: the anterior lateral motor cortex (ALM) and the primary visual cortex (VISp). We used the Allen’s metadata to parse cells by clusters representing major excitatory and inhibitory classes that are common to both ALM and VISp. We then performed two types of pairwise differential gene expression analysis: (1) between different neuronal classes within the same brain region (ALM or VISp), and (2) between the same neuronal class in ALM and VISp. We filtered our results for differentially expressed genes related to circuit connectivity and developed a novel bioinformatic approach to determine the sets uniquely enriched in each neuronal class in ALM, VISp, or both. This analysis provides an organized set of genes that may regulate synaptic connectivity and physiology in a cell-type-specific manner. Furthermore, it identifies candidate mechanisms for circuit organization that are conserved across functionally distinct cortical regions or that are region dependent. Finally, we used the SFARI Human Gene Module to identify genes from this analysis that are related to risk for autism spectrum disorder (ASD). Our analysis provides clear molecular targets for future studies to understand neocortical circuit organization and abnormalities that underlie autistic phenotypes.

## Introduction

In the neocortex, excitatory projection neurons and inhibitory interneurons can be grouped into major classes that are found across functionally distinct regions. Excitatory classes are defined by their long-range axonal projection: intratelencephalic (IT), pyramidal tract (PT), or corticothalamic (CT) (Harris and Shepherd, 2015). Interneuron classes are defined by the expression of molecular markers such as parvalbumin (PV), somatostatin (SST), and vasoactive intestinal peptide (VIP) (Tremblay et al., 2016). Importantly, recent work suggests that neurons from these classes form microcircuit motifs that may be repeated across the cortex. For example, in prefrontal, motor, and visual cortices, IT cells from synaptic connections onto PT cells that are largely unreciprocated. (Morishima and Kawaguchi, 2006; Brown and Hestrin, 2009b; Kiritani et al., 2012). Among interneurons, VIP+ cells preferentially inhibit neighboring SST+ cells in primary sensory and prefrontal cortices (Lee et al., 2013; Pfeffer et al., 2013; Pi et al., 2013; Karnani et al., 2016). Furthermore, in deep cortical layers, PV+ interneurons preferentially target PT cells relative to IT cells (Lee et al., 2014; Wu et al., 2016; Ye et al., 2016), and VIP+ interneurons are targeted by IT cells but not PT cells (Wester et al., 2019). Thus, cell class appears to serve an important role in organizing canonical circuits for fundamental computations throughout the cortex (Douglas and Martin, 2004; Harris and Shepherd, 2015; Luo, 2021).

Recent single-cell RNA-sequencing studies have revolutionized our understanding of neocortical cell types and provide candidate molecular targets to study their synaptic connections (Zeisel et al., 2015; Tasic et al., 2016, 2018; Paul et al., 2017; Saunders et al., 2018; Yao et al., 2021). These data reveal many subtypes of neurons within each major class, setting a foundation to investigate circuits with higher precision (Huang and Paul, 2019). However, they also raise questions regarding how to define the major classes in different cortical regions, which has important implications for understanding circuit organization. Specifically, excitatory classes vary in their transcriptomic profiles from the rostral to caudal poles (Saunders et al., 2018; Tasic et al., 2018; Yao et al., 2021). Thus, PT cells in primary visual cortex and prefrontal cortex may be best matched, but still categorized as different cell-types. In contrast, the transcriptomic profiles of interneuron classes are consistent throughout the cortex (Saunders et al., 2018; Tasic et al., 2018; Yao et al., 2021). This suggests that circuit motifs involving excitatory classes may be more regionally specialized than those involving interneurons. However, the extent to which the connectivity patterns of excitatory or inhibitory neurons can be generalized is unclear and an area of active research (Brown and Hestrin, 2009a; Huang and Paul, 2019; Luo, 2021). Indeed, recent work argues that circuits involving major interneuron classes are also dependent on region (Pouchelon et al., 2021). Thus, understanding how intrinsic class properties and areal cues guide the assembly of circuits remains a challenge.

Resolving these issues may be important for understanding and treating neuropsychiatric disorders. For example, it is hypothesized that an imbalance in the ratio of excitation to inhibition (E/I) within cortical circuits contributes to autism spectrum disorder (ASD) (Sohal and Rubenstein, 2019). Importantly, impairments that characterize ASD range from aberrant social behavior (entailing prefrontal circuits) (Yizhar et al., 2011) to sensory processing deficits (entailing visual cortical circuits) (Del Rosario et al., 2021). Thus, a tantalizing extension of this hypothesis is that in some instances ASD results from disruption of a canonical circuit motif that is repeated across functionally distinct regions. Alternatively, if circuit motifs in each region are unique, this may complicate therapeutic strategies. Several groups are now using monogenetic mouse models to investigate the contributions of different classes of excitatory and inhibitory neurons to the emergence of ASD phenotypes (Brumback et al., 2018; Mossner et al., 2020; Smith et al., 2020). However, clinically relevant mechanisms for synaptic connectivity, and if they are region specific, are unclear.

Here, we analyzed single-cell RNA-sequencing data from the Allen Institute for Brain Science (Tasic et al., 2018) to investigate molecular signatures of circuit organization in major neuronal classes across the neocortex. We used data from two cortical regions at opposite ends of the rostral-caudal axis to compare extremes in expression profiles: the motor planning anterior lateral motor cortex (ALM) and sensory processing primary visual cortex (VISp). Our analysis identifies synaptic genes that are enriched in select classes of excitatory and inhibitory cells. Furthermore, we determine which of these patterns are conserved in ALM and VISp, or unique to each region. Finally, we highlight classes that likely harbor specific ASD risk genes across the cortex and thus may be candidates for understanding constellations of conditions.

## Results

### Transcriptomic profiles of both excitatory and inhibitory neurons are primarily differentiated by class rather than brain region

**FIGURE 1:**
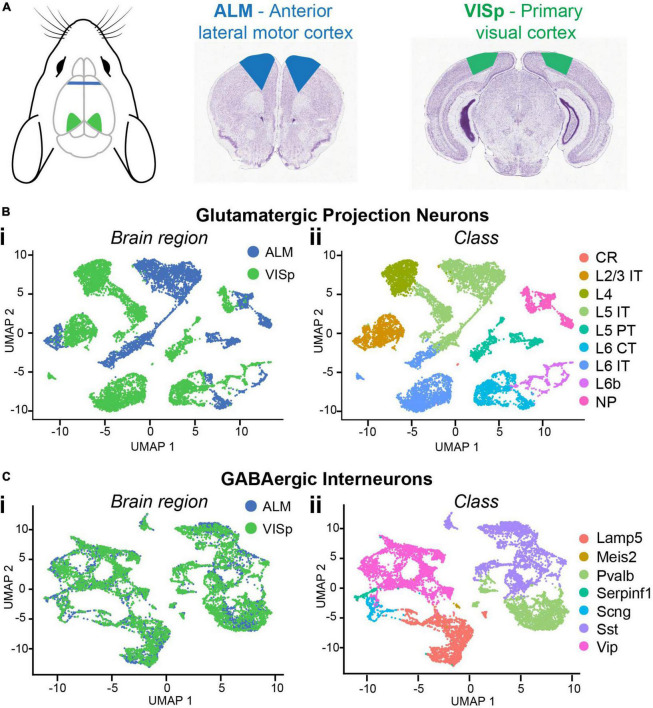
Single-cell RNA-seq data for major neuronal classes in ALM and VISp. **(A)** Anatomical locations of ALM (blue) and VISp (green) with corresponding coronal sections. Mouse cartoon modified from SciDraw (doi: 10.5281/zenodo.3925903). **(B)** UMAP dimensionality reduction for glutamatergic cells color-coded using metadata for brain region **(Bi)** or class **(Bii)**. **(C)** UMAP dimensionality reduction for GABAergic cells color-coded using metadata for brain region **(Ci)** or class **(Cii)**. L2/3 IT, L5 IT, L5 PT, L6 CT, Pvalb, Sst, and Vip clusters were selected for downstream analysis. CR, Cajal–Retzius; IT, intratelencephalic; PT, pyramidal tract; CT, corticothalamic; NP, near-projecting; Pvalb, parvalbumin; Sst, somatostatin; Vip, vasoactive intestinal peptide. Coronal sections adapted from the Allen Mouse Brain Atlas (https://mouse.brain-map.org/static/atlas).

**FIGURE 2:**
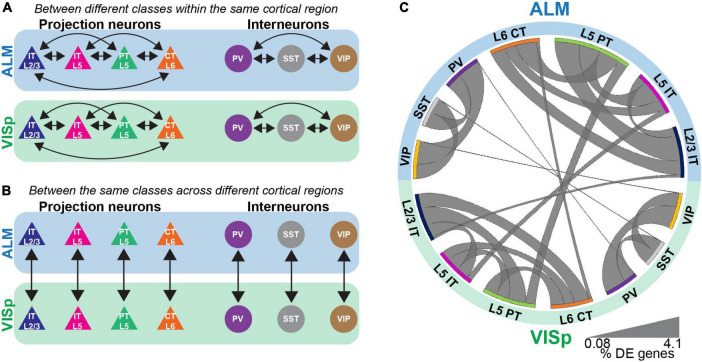
Differential expression tests for major neuronal classes either within or between cortical regions. **(A)** Pairwise differential expression tests conducted between excitatory or inhibitory neuronal classes in ALM or VISp. **(B)** Pairwise differential expression tests conducted between the same neuronal classes across ALM or VISp. **(C)** Circos plot summary of the percentage of differentially expressed genes found in each pairwise comparison. Classes are listed along the circumference of the plot in their respective brain region (blue - ALM; green - VISp). Bands connect classes that were compared in a pairwise differential expression test. Band thickness represents the proportion of differentially expressed genes in each test: thinner bands indicate a small percentage of distinguishing genes and thicker bands indicate a larger percentage of distinguishing genes.

To compare neuronal subtypes between two functionally distinct cortical regions, we downloaded the Allen Institute’s single-cell RNA-sequencing datasets of adult mouse cortical neurons collected from the anterior lateral motor cortex (ALM) and primary visual cortex (VISp) (Tasic et al., 2018; Figure 1A). These include metadata that define each cell’s brain region (ALM or VISp), neurotransmitter (glutamatergic or GABAergic), and major class (e.g., L2/3 IT for excitatory neurons or Pvalb for inhibitory interneurons). We loaded these data into the R toolkit Seurat (Stuart et al., 2019) and ran the non-linear dimensionality reduction algorithm UMAP for unbiased clustering of excitatory projection neurons and inhibitory interneurons (Figures 1B,C). We used the metadata to label each cell according to its brain region (Figures 1Bi,Ci) or major class (Figures 1Bii,Cii). As expected, this reproduced the findings of Tasic et al. (2018) that excitatory neurons cluster according to both brain region and class (Figure 1B) but interneurons cluster only by class (Figure 1C). This suggests that major classes of excitatory neurons (e.g., L2/3 IT) are not conserved across cortical regions but are distinct in ALM and VISp (Tasic et al., 2018). However, relative positions of clusters in UMAP space do not indicate the magnitude of differences between cell-types. Thus, we performed differential gene expression analyses to compare the number of genes that distinguish neuronal classes within and across brain regions (Figures 2A,B).

The Allen’s ALM and VISp datasets include a total of 25,481 neuronal and non-neuronal cells that were profiled from adult mice ages P51, P53–P59, and P63–P91 (Tasic et al., 2018). For our analyses, we chose a subset of major excitatory and inhibitory neuronal classes that are shared between ALM and VISp and are relatively well studied (Harris and Shepherd, 2015; Tremblay et al., 2016). For excitatory cells, these included layer 2/3 intratelencephalic (L2/3 IT), layer 5 intratelencephalic (L5 IT), layer 5 pyramidal tract (L5 PT), and layer 6 corticothalamic (L6 CT). For inhibitory cells, these included those expressing parvalbumin (PV), somatostatin (SST), or vasoactive intestinal peptide (VIP). The number of neurons included in each class and brain region considered in our analysis is presented in Table 1. We conducted two types of analyses: (1) pairwise differential expression tests between different excitatory or inhibitory classes within the same cortical region (ALM or VISp) (Figure 2A) and (2) pairwise differential expression tests between the same classes across ALM and VISp (Figure 2B). For each pairwise test, we counted the number of differentially expressed genes and normalized it to the total number compared (Supplementary Table 1, “Circos Table,” “All Genes” columns). This allowed us to determine the proportions of differentially expressed genes among all the pairwise tests both within and across brain regions, which we visualized using a Circos plot (Krzywinski et al., 2009; Figure 2C). For both excitatory and inhibitory cells, we found that each major class is more similar between brain regions than between other classes within the same brain region. Thus, even though excitatory neurons cluster by brain region (Figure 1B), class identity best distinguishes them. Interestingly, we also found differences between major inhibitory classes across ALM and VISp, suggesting that regional cues also influence their differentiation (Pouchelon et al., 2021). Our results agree with previous work that major excitatory and inhibitory classes are primarily conserved across diverse cortical regions when considering transcriptomic profiles (Tasic et al., 2018; Yao et al., 2021).

**TABLE 1:** Number of cells analyzed for each neuronal class and cortical region.

Class	ALM	VISp
L2/3 IT	325	982
L5 IT	2401	880
L5 PT	368	544
L6 CT	350	960
PV	896	1337
SST	1139	1741
VIP	1224	1728

### Genes relevant to synaptic connectivity are primarily differentiated by class rather than brain region

**FIGURE 3:**
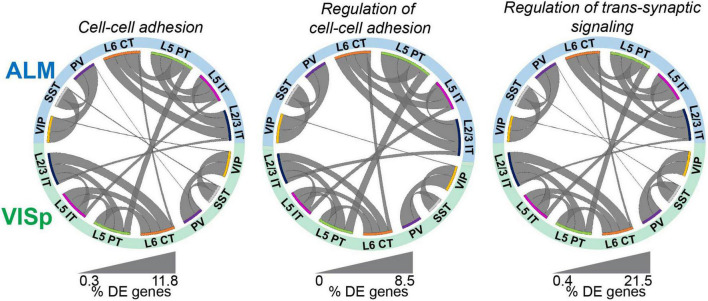
Circuit-related genes are primarily differentiated by class rather than cortical region. Circos plot summary of the percentage of differentially expressed circuit-related genes found in each pairwise comparison. Circuit-related genes were identified using PANTHER gene ontology labels “cell-cell adhesion”, “regulation of cell-cell adhesion”, and “regulation of trans-synaptic signaling”. Classes are listed along the circumference of the plot in their respective brain region (blue - ALM; green - VISp). Bands connect classes that were compared in a pairwise differential expression test. Band thickness represents the proportion of differentially expressed genes in each test: thinner bands indicate a small percentage of distinguishing genes and thicker bands indicate a larger percentage of distinguishing genes. No genes involved in Regulation of cell-cell adhesion were differentially expressed between SST or PV cells across regions.

Although major neuronal classes largely share transcriptomic profiles between ALM and VISp, these two cortical regions are functionally distinct. Thus, we next asked if genes related to synaptic connectivity and circuit organization are also shared for each neuronal class across the cortex. We filtered our differential expression results using the gene ontology (GO) software PANTHER 16.0 (Mi et al., 2021) to select genes labeled by the terms “Cell-cell adhesion,” “Regulation of cell-cell adhesion,” and “Regulation of trans-synaptic signaling.” These were chosen because they include genes related to the formation, maintenance, and plasticity of synaptic connections among distinct neuronal classes (Fuccillo et al., 2015; Földy et al., 2016; Chowdhury et al., 2021). Throughout, we refer to these as “circuit-related” genes. For all three GO terms, we found lower proportions of differentially expressed circuit-related genes between the same class in ALM and VISp than between different classes, regardless of brain region (Figure 3 and Supplementary Table 1, “Circos Table”). This matches the pattern observed for differential expression of all transcripts (Figure 2C). Thus, these analyses suggest that despite the functional distinction of ALM and VISp, gene expression profiles that define cell-types and their markers for circuit organization are largely conserved across the neocortex.

### Identifying the circuit-related genes that are differentially expressed between classes reveals that most are region-specific for excitatory projection neurons

**FIGURE 4:**
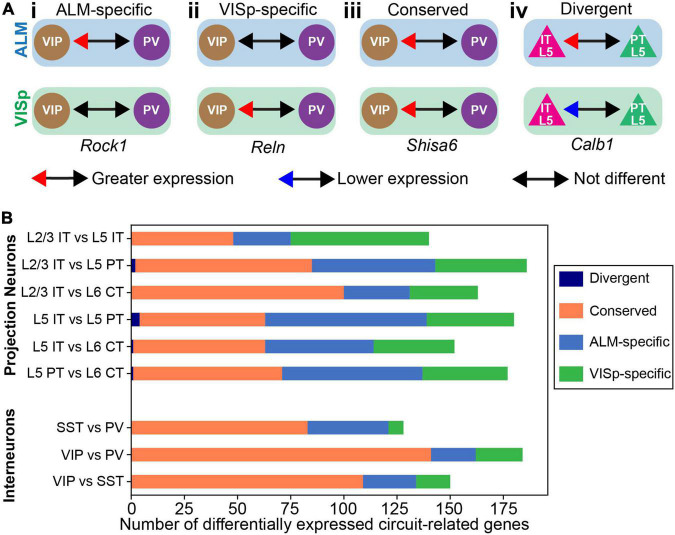
Categorization of circuit-related genes that are differentially expressed between neuronal classes within ALM or VISp. **(A)** Examples of differential expression test results used to categorize circuit-related genes as: ALM-specific **(Ai)**, VISp-specific **(Aii)**, Conserved **(Aiii)**, or Divergent **(Aiv)**. The genes and results are real examples from the analysis. **(B)** Stacked bar plot of the total number of circuit-related genes in each category for every pairwise differential expression test between classes. “Divergent” genes were only observed in L2/3 IT vs L5 PT; L5 IT vs L5 PT; L5 IT vs L6 CT; and L5 PT vs L6 CT comparisons.

The above analyses simply consider the proportions of differentially expressed genes to describe general trends in the data. Thus, by percentage most circuit-related genes are differentially expressed between neuronal classes within ALM and VISp rather than between the same classes across these cortical regions (Figure 3). However, this analysis does not consider if the genes that are differentially expressed between neuronal classes within ALM and VISp are different sets. This could obscure the true number of genes that provide region-specific instructions for circuit organization. Thus, for each differential expression test between neuronal classes within ALM or VISp, we categorized the result as either (1) ALM-specific, (2) VISp-specific, (3) conserved, or 4) divergent (Figure 4A). For example, *rock1* is differentially expressed between VIP and PV cells in ALM but not VISp (ALM-specific, Figure 4Ai). Similarly, *reln* is differentially expressed between VIP and PV cells in VISp but not ALM (VISp-specific, Figure 4Aii). Importantly, for a gene that is differentially expressed between the same pair of neuronal classes in both ALM and VISp, it is necessary to consider which class had greater expression in each comparison. For example, *shisa6* exhibits greater expression in VIP cells relative to PV cells in both ALM and VISp (conserved, Figure 4Aiii). However, *calb1* exhibits greater expression in L5 IT cells relative to L5 PT cells in ALM but this relationship is reversed in VISp (divergent, Figure 4Aiv). We developed an algorithm to classify differential expression test results in this manner (described in Methods) and implemented it in Python as “DE_Collapser”. This code is available on GitHub (see Data Availability).

In Figure 4B, we plotted the total number of circuit-related genes in each category that demonstrated significant differential expression between neuronal classes. Importantly, genes in the “divergent” category were rare and only observed in a subset of tests between excitatory projection neurons (only 7 genes in 4 tests). Thus, the analysis from our differential expression tests yielded few results that would require more complicated interpretations by considering each brain region separately. As expected, for inhibitory interneurons most differential expression test results were conserved between ALM and VISp. This is consistent with the observation that interneuron expression profiles do not cluster by brain region (Figure 1C) and our Circos plots of proportions of differentially expressed genes within and across cortical regions (Figures 2C, 3). However, we identified several genes for which differential expression results were ALM- or VISp-specific and consider them in detail in below (Figures 8, 9). Strikingly, for excitatory neurons we found that more than half of the circuit-related genes in each comparison were exclusive to ALM or VISp (except for the L2/3 IT vs. L6 CT comparison). This appears incongruent with our above results that excitatory classes have a lower proportion of differentially expressed circuit-related genes across brain regions (Figure 3). However, we emphasize that this analysis is unique from Figures 2, 3. In Figure 4, we consider the *identity* of circuit-related genes that are differentially expressed between neuronal classes *within* each brain region (as in Figure 2A) to determine if the gene sets and their differential expression results overlap. This reveals that for excitatory projection neurons most circuit-related genes that distinguish classes within ALM and VISp are exclusive to each region.

**FIGURE 5:**
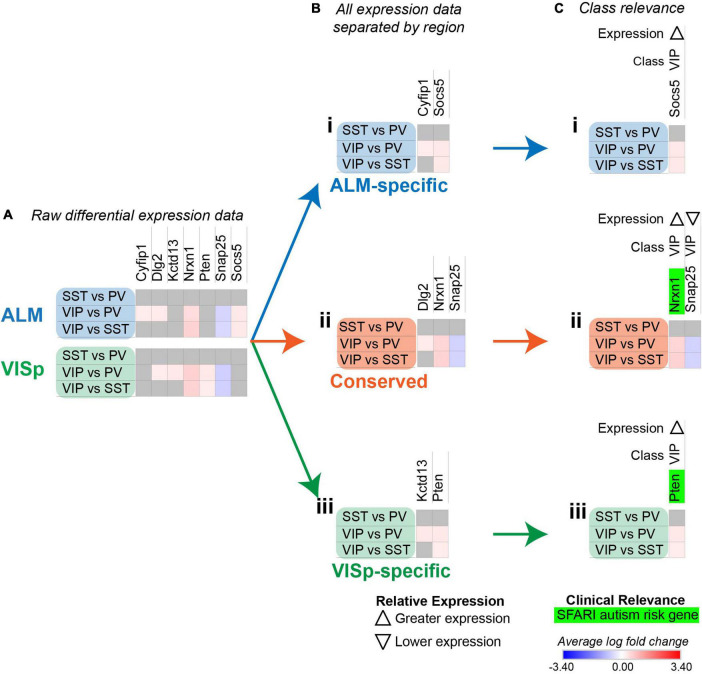
Strategy to determine circuit-related genes relevant to specific neuronal classes. Each row (e.g., SST vs. PV) presents the results of differential expression tests between neuronal classes. Differential expression results are always relative to the first neuronal class in a comparison (e.g., SST in ‘SST vs PV’). Gray indicates no significance in differential expression. Shades of red and blue indicate the log fold change of average expression for tests that were statistically significant. **(A)** Examples of differential expression rests results for seven circuit-related genes in interneuron classes in ALM and VISp. **(B)** Separation of genes from *(A)* into ALM-specific **(Bi),** Conserved **(Bii)**, and VISp-specific **(Biii)** subsets. **(C)** Class-relevant genes from *(B)* identified by their consistent differential expression results among all class tests. For each gene, we provide a class-relevant label and an arrow indicating whether it is expressed higher or lower relative to other classes. Class-relevant genes listed on the SFARI Human Gene Module implicated in autism were highlighted green.

**FIGURE 6:**
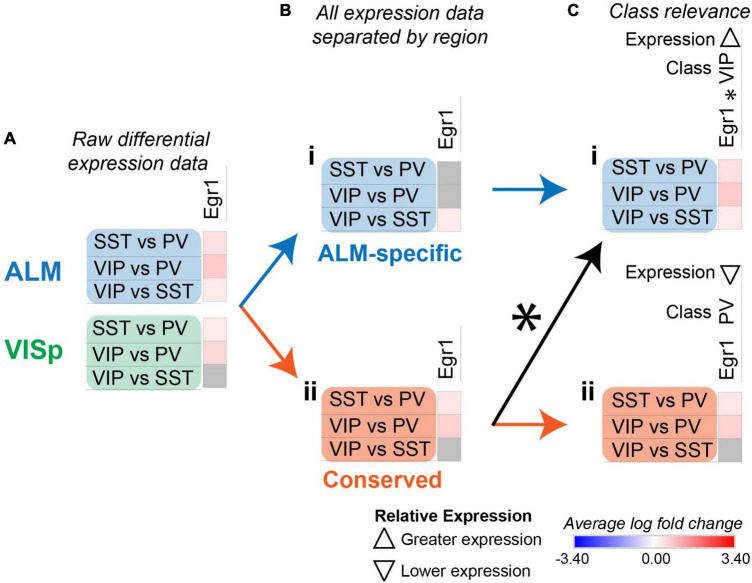
Strategy to identify class-relevant genes that describe both conserved and region-specific relationships. Each row (e.g., SST vs. PV) presents the results of differential expression tests between neuronal classes. Differential expression results are always relative to the first neuronal class in a comparison (e.g., SST in ‘SST vs. PV’). Gray indicates no significance in differential expression. Shades of red and blue indicate the log fold change of average expression for tests that were statistically significant. **(A)** Example differential expression test results for *egr1* in both ALM and VISp. **(B)** Separation of differential expression results into subsets that are ALM-specific **(Bi)** and conserved in both ALM and VISp **(Bii). (C)** Annotation of *erg1* using an asterisk (*) to denote that a subset of ALM-specific differential expression results **(Ci)** is also found within the set labeled as “Conserved” **(Cii)**. Note that for *erg1*, the relevant class and relative expression-level are different in *Ci* and *Cii* because the combinations of differential expression results among neuronal classes are unique. Up and down arrows denote whether expression is higher or lower relative to the other classes.

**FIGURE 7:**
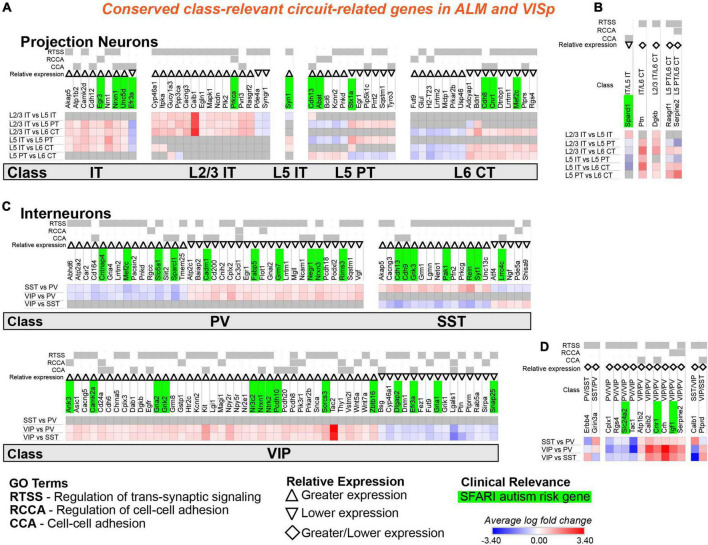
Circuit-related genes that are relevant to the same neuronal classes in ALM and VISp. Each row (e.g., L2/3 IT vs. L5 IT) presents the results of differential expression tests between neuronal classes. Differential expression results are always relative to the first neuronal class in a comparison (e.g., L2/3 IT in ‘L2/3 IT vs. L5 IT’). Gray indicates no significance in differential expression. Shades of red and blue indicate the log fold change of average expression for tests that were statistically significant. Up and down arrows denote whether expression is higher or lower relative to the other classes. The diamond symbol in **(B,D)** indicates that a gene is relevant to two different classes: it is equivalent to an up arrow for the first gene and a down arrow for the second. For example, in **(D)** the PV class has greater expression of Erbb4 relative to both VIP and SST classes, and the SST class has lower expression relative to both VIP and PV classes. **(A)** Circuit-related genes relevant to single classes of excitatory projection neurons. We created the new class “IT” for genes that were relevant to both L2/3 IT and L5 IT classes. **(B)** Circuit-related genes relevant to multiple classes of excitatory projection neurons. **(C)** Circuit-related genes relevant to single classes of inhibitory interneurons. **(D)** Circuit-related genes relevant to multiple classes of inhibitory interneurons. CCA = cell-cell adhesion; RCCA = regulation of cell-cell adhesion; RTSS = regulation of trans-synaptic signaling.

**FIGURE 8:**
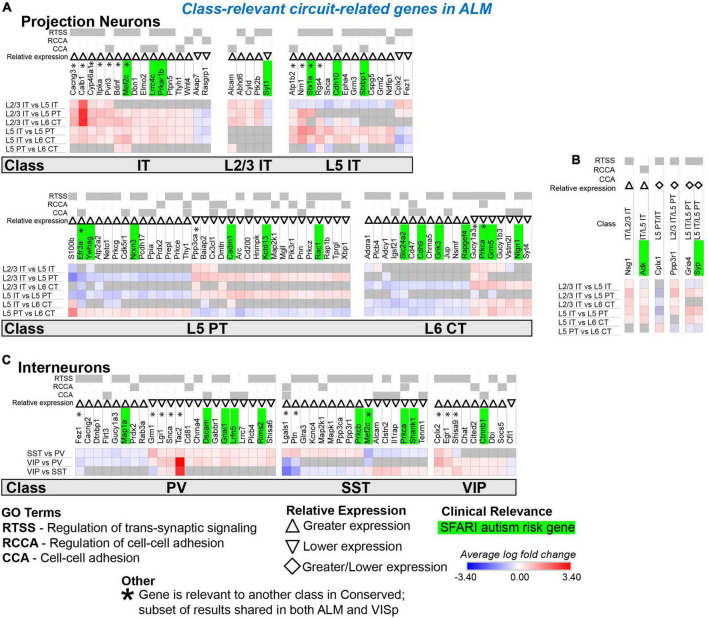
Circuit-related genes only relevant to neuronal classes in ALM. Each row (e.g., L2/3 IT vs. L5 IT) presents the results of differential expression tests between neuronal classes. Differential expression results are always relative to the first neuronal class in a comparison (e.g., L2/3 IT in ‘L2/3 IT vs. L5 IT’). Gray indicates no significance in differential expression. Shades of red and blue indicate the log fold change of average expression for tests that were statistically significant. Up and down arrows denote whether expression was higher or lower relative to the other classes. **(A)** Circuit-related genes relevant to single classes of excitatory projection neurons. We created the new class “IT” for genes that were relevant to both L2/3 IT and L5 IT classes. **(B)** Circuit-related genes relevant to multiple classes of excitatory projection neurons. The diamond symbol indicates that a gene is relevant to two different classes: it is equivalent to an up arrow for the first gene and a down arrow for the second. For example, the L5 PT class has greater expression of Cplx1 relative to the other classes, and the IT class has lower expression relative to the L5 PT and L6 CT classes. **(C)** Circuit-related genes relevant to single classes of inhibitory interneurons. CCA = cell-cell adhesion; RCCA = regulation of cell-cell adhesion; RTSS = regulation of trans-synaptic signaling.

**FIGURE 9:**
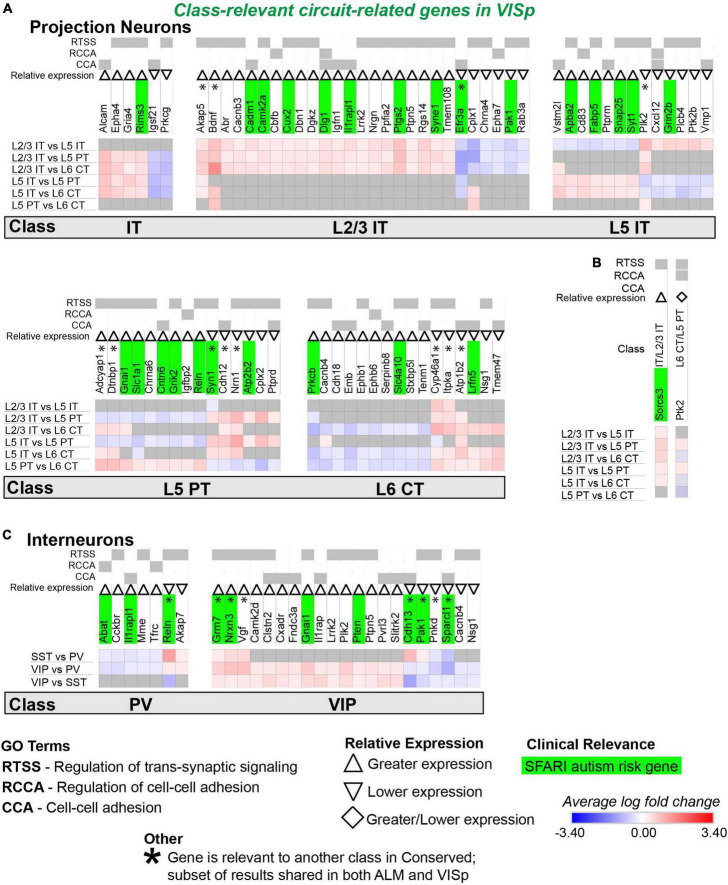
Circuit-related genes only relevant to neuronal classes in VISp. Each row (e.g., L2/3 IT vs. L5 IT) presents the results of differential expression tests between neuronal classes. Differential expression results are always relative to the first neuronal class in a comparison (e.g., L2/3 IT in ‘L2/3 IT vs. L5 IT’). Gray indicates no significance in differential expression. Shades of red and blue indicate the log fold change of average expression for tests that were statistically significant. Up and down arrows denote whether expression was higher or lower relative to the other classes. **(A)** Circuit-related genes relevant to single classes of excitatory projection neurons. We created the new class “IT” for genes that were relevant to both L2/3 IT and L5 IT classes. **(B)** Circuit-related genes relevant to multiple classes of excitatory projection neurons. The diamond symbol indicates that a gene is relevant to two different classes: it is equivalent to an up arrow for the first gene and a down arrow for the second. For example, and the L6 CT class has greater expression of Ptk2 relative to the other classes, and the L5 PT class has lower expression relative to the other classes. **(C)** Circuit-related genes relevant to single classes of inhibitory interneurons. CCA = cell-cell adhesion; RCCA = regulation of cell-cell adhesion; RTSS = regulation of trans-synaptic signaling.

### The results from individual differential expression tests can be combined to identify circuit-related genes relevant to each major neuronal class

In the above analysis, we found 1,452 significant differential expression test results for 394 circuit-related genes that were exclusive to ALM, exclusive to VISp, or conserved between cortical regions (Figure 4B and Supplementary Table 1 “StackedBar Table”). This makes it challenging to derive practical biological insights from these data. Thus, we developed a novel bioinformatic approach to narrow our results to a tractable set of genes that have a high likelihood to play key roles in cortical circuit organization. Our strategy identified circuit-related genes for which differential expression results were consistent across all tests for each neuronal class. As an example, we consider a subset of seven circuit-related genes with significant differential expression among inhibitory neuronal classes in Figure 5. *Cyfip1* has greater expression in VIP cells than PV cells in ALM, but not in VISp (Figure 5A); thus, we classified this gene as ALM-specific (Figure 5Bi). *Dlg2* has greater expression in VIP cells than PV cells in *both* ALM and VISp (Figure 5A); thus, we classified this gene as Conserved (Figure 5Bii). Finally, *kctd13* has greater expression in VIP cells than PV cells in VISp, but not in ALM (Figure 5A); thus, we classified this gene as VISp-specific (Figure 5Biii). For these three genes, differential expression between SST and PV cells is not significantly different; thus, we would predict greater expression in VIP cells relative to SST cells for each. However, this was not the case (Figure 5A), which renders the biological relevance of these results unclear. In contrast, *nrxn1*, *pten*, *snap25*, and *socs5*, all demonstrate consistent differential expression results among all three tests (Figure 5A). Thus, we sorted these genes as ALM-specific (*socs5*), VISp-specific (*pten*), or Conserved (*nrxn1* and *snap25*), as above (Figure 5B). We then kept these four genes for further analysis, and discarded *cyfip1*, *dlg2*, and *kctd13* (Figure 5C). Next, we defined *nrxn1*, *pten*, *snap25*, and *socs5* as “class-relevant” to VIP cells and denoted whether expression was higher or lower relative to the other classes with up and down arrows, respectively (see Figure 5Cii). Finally, dysregulation of circuit-related genes directly contributes to neurological disorders (Chowdhury et al., 2021). Thus, we highlighted in green candidate risk genes for autism using the SFARI Human Gene Module^1^ (Figure 5C). The algorithm to classify genes as “class-relevant” according to cortical region was implemented in Python as “SCID” and described in the Methods section. This code is available on GitHub (see Data Availability).

For some class-relevant genes, a subset of their differential expression results was shared between ALM and VISp, and a subset was exclusive to one cortical region. As an example, we include the analysis of *egr1* in Figure 6. *Erg1* demonstrates significant differential expression in all three tests between interneuron classes in ALM, but in only two tests in VISp (Figure 6A). Thus, in ALM *erg1* has greater expression in VIP cells relative to SST cells (Figure 6Bi), while in both ALM and VISp *erg1* has greater expression in SST cells relative to PVs cells and in VIP cells relative to PV cells (Figure 6Bii). These differences are important for interpreting the relevance of *erg1* expression among neuronal classes. In ALM, we define *erg1* as having greatest expression in VIP cells relative to PV and SST cells (Figure 6Ci). However, we indicate with an asterisk (*) that a subset of these results is conserved in VISp, where we define *erg1* as having lower expression in PV cells relative to VIP and SST cells (Figure 6Cii). The algorithm to identify class-relevant genes that share a subset of their differential expression results between cortical regions was implemented in Python as “CortexSCID” and described in the Methods section. This code is available on GitHub (see Data Availability).

In summary, our strategy combined pairwise differential expression test results to relate each circuit-related gene to a major neuronal class. This allowed us to create an organized and tractable list of genes that are candidates to mediate synaptic connections between specific neuronal classes across the cortex or dependent on cortical region (Figures 7-9). Of the 394 circuit-related genes in the ALM-specific, VISp-specific, and Conserved subsets, 71% (280) mapped to at least one class. Furthermore, 26% of these are risk genes for autism and may inform future studies investigating the circuit mechanisms of this disorder. We describe these data in greater detail below and provide a searchable spreadsheet that identifies each gene-class relationship (Supplementary Table 2).

### Most circuit-related genes identified as conserved are class-relevant to inhibitory interneurons

Our analysis identified 57 circuit-related genes relevant to excitatory neuronal classes that were conserved in both ALM and VISp (Figures 7A,B). A subset of 9 genes was relevant to both L2/3 IT and L5 IT classes; thus, we created a new class “IT” for these genes (Figure 7A). Indeed, most circuit-related genes that were conserved among excitatory neurons in both ALM and VISp were relevant to the L2/3 IT, L5 IT, and combined IT classes. Only a small number of genes (5) were relevant to other combinations of multiple classes (e.g., L2/3 IT and L6 CT) in both ALM and VISp (Figure 7B). Interestingly, more than twice as many genes (124) were conserved for interneuron classes compared to excitatory classes (Figures 7C,D). Furthermore, most of these genes were unique to interneurons; only 23 genes were identified as class-relevant in both the excitatory and inhibitory populations. Thus, the mechanisms that regulate synaptic connections in a class-specific manner may be mostly distinct between excitatory and inhibitory cell types. Among interneurons, most were relevant to VIP cells and distinguished them from PV and SST cells (Figure 7C). Thus, the mechanisms underlying the formation of disinhibitory circuits appear to be conserved across cortical regions. Like excitatory neurons, only a small number of genes (14) were relevant to combinations of multiple interneuron classes (e.g., PV and SST) in both ALM and VISp (Figure 7D). Finally, SFARI autism risk genes were distributed uniformly among all excitatory and inhibitory classes and represented 23% and 28% of the total genes for each group, respectively. Thus, we could not identify a specific set of neuronal classes that may be uniquely vulnerable in autism. In summary, we identified circuit-related genes that are conserved between ALM and VISp for each neuronal class and may mediate molecular mechanisms underlying stereotyped “canonical circuits” found across the cortex (Kubota, 2014; Harris and Shepherd, 2015; Gutman-Wei and Brown, 2021). Furthermore, many of them are candidates to disrupt the balance between excitation and inhibition throughout the cortex in developmental disorders (Sohal and Rubenstein, 2019).

### Most circuit-related genes that are identified as specific to classes in ALM or VISp are in excitatory projection neurons

We next evaluated circuit-related genes that are relevant to neuronal classes exclusively in ALM (Figure 8) or VISp (Figure 9). In ALM, we found 92 genes relevant to excitatory classes (Figure 8A), 15 of which shared a subset of differential expression results that were also relevant to classes in VISp (Figure 8A, genes denoted by * are also found in Figure 7A). Only a small number of genes (6) were relevant to combinations of multiple classes (e.g., L5 IT and L5 PT) exclusively in ALM (Figure 8B). For interneurons, we found 47 class-relevant genes (Figure 8C), 11 of which shared a subset of differential expression results that were also relevant to classes in VISp (Figure 8C, genes denoted by * are also found in Figure 7C). Like our analyses of conserved genes (Figure 7), few were shared between excitatory and inhibitory classes (12 total). However, in contrast to conserved genes, most were class relevant to excitatory cells. Furthermore, among excitatory cells most genes were relevant specifically to the IT classes. Our analysis of genes that were class relevant in VISp yielded similar results (Figure 9). In VISp, we found 79 genes relevant to excitatory classes (Figure 9A), 12 of which shared a subset of differential expression results that were also relevant to classes in ALM (Figure 9A, genes denoted by * are also found in Figure 7A). Only 2 genes were relevant to combinations of multiple classes (e.g., L5 PT and L6 CT) exclusively in VISp (Figure 9B). For interneurons, we found 28 class-relevant genes (Figure 9C), 8 of which shared a subset of differential expression results that were also relevant to classes in ALM (Figure 9C, genes denoted by * are also found in Figure 7C). Like in ALM, most genes in VISp were relevant to excitatory IT-type classes, and few overlapped between excitatory and inhibitory cells (9 total).

In summary, we found that most of the class-relevant circuit-related genes that are dependent on cortical region are found in excitatory neurons. Furthermore, most of these are relevant to IT-type cells and many are candidate risk genes for autism. These data agree with previous work in humans that IT-type cells may be a key contributor to autistic phenotypes (Parikshak et al., 2013). However, our results highlight that the development of therapies to target excitatory cells may not work uniformly across the cortex.

### A small set of genes are uniquely enriched in ALM or VISp

**FIGURE 10:**
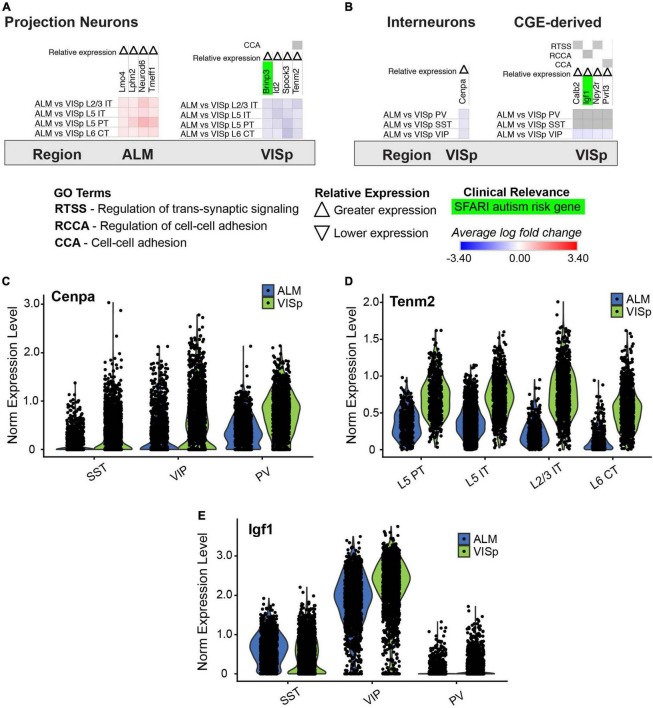
Genes that have biased expression to ALM or VISp for all excitatory or inhibitory neuronal classes. Each row (e.g., ALM vs. VISp L2/3 IT) presents the results of differential expression tests for a neuronal class between cortical regions. Differential expression results are always relative to the first cortical region in a comparison (e.g., ALM in ‘ALM vs. VISp L2/3 IT’). Gray indicates no significance in differential expression. Shades of red and blue indicate the log fold change of average expression for tests that were statistically significant. Up and down arrows denote whether expression was higher or lower relative to the other cortical region. **(A)** Genes with biased expression between ALM or VISp for excitatory projection neuron classes. Note that only 1 gene, *tenm2*, is circuit-related. **(B)** Genes with biased expression between ALM or VISp for inhibitory interneuron classes. (*Left*) Only 1 gene, *cenpa*, had biased expression among all classes. (*Right*) Only 4 circuit-related genes demonstrated biases in expression when comparing VIP to PV/SST classes. **(C)** Violin plots of *cenpa* single-cell expression for inhibitory interneuron classes in ALM and VISp. **(D)** Violin plots of *tenm2* single-cell expression for excitatory neuronal classes in ALM and VISp. **(E)** Violin plots of *igf1* single-cell expression for inhibitory interneuron classes in ALM and VISp.

Finally, we analyzed the differential expression results for each class between ALM and VISp (Figure 10; see Figure 2B for schematic). We hypothesized there are genes that are uniquely expressed between these functionally distinct and spatially distant cortical regions. Thus, we filtered genes that demonstrated consistent differential expression results for all neuronal classes between ALM and VISp. Strikingly, this analysis identified few genes. Among excitatory classes, only 4 genes were biased to ALM (*lmo4*, *lphn2*, *neurod6*, and *tmeff1*) and only 4 were biased to VISp (*brinp3*, *id2*, *spock3*, *tenm2*) (Figure 10A). Only 1 of these genes, *tenm2*, was circuit-related (Figures 10A,D). Among inhibitory classes, we found only 1 gene (*cenpa*) that was biased to VISp, but it was not circuit-related according to gene ontology (but see Discussion section) (Figures 10B,C). Finally, PV and SST interneurons share their developmental origin from the medial ganglionic eminence (Xu et al., 2004; Butt et al., 2005), but VIP interneurons are derived from the caudal ganglionic eminence (Lee et al., 2010; Miyoshi et al., 2010). Thus, we asked if there are circuit-related genes that distinguish interneurons in ALM and VISp dependent on embryonic origin. We found none for PV/SST classes and only 4 such genes (*calb2*, *igf1*, *npy2r*, and *pvrl3*) for the VIP class (Figures 10B,E). Of note, a previous study showed that *igf1* expression in VIP interneurons regulates experience-dependent plasticity in primary visual cortex (Mardinly et al., 2016). In summary, surprisingly few genes collectively distinguish excitatory and inhibitory neuronal classes between the rostral and caudal poles of the cortex.

## Discussion

Recent single cell RNA-sequencing data from multiple groups suggest that excitatory neuronal cell types are distinct in different regions of the neocortex (Saunders et al., 2018; Tasic et al., 2018; Bhattacherjee et al., 2019; Yao et al., 2021), which has important implications for circuit organization. Here, we mined data from the Allen Institute (Tasic et al., 2018) to investigate molecular signatures of synaptic connectivity among major classes of excitatory and inhibitory neurons between two spatially distant and functionally distinct cortical regions. An advantage of the Allen Institute data is that major neuronal classes were enriched using transgenic mice and viral approaches prior to sequencing. Thus, using provided metadata for each neuron, we analyzed best-matched neuronal classes between ALM and VISp. Our unique approach combined results from multiple differential expression tests to assign circuit-related genes to specific neuronal classes. Furthermore, we directly compared the sets of class-relevant genes identified within ALM and VISp. This allowed us to organize genes according to their enrichment in major classes and determine if their expression within each class was shared between ALM and VISp or unique to each. Thus, our study provides insight into molecular mechanisms of circuit organization that are either conserved or region-dependent and identifies clear targets for future studies.

### Regional differentiation of neuronal classes and implications for circuit organization

Excitatory projection neuron progenitors in the developing dorsal telencephalon are organized as a protomap of cortical regions (Rakic, 1988; Elsen et al., 2013; Greig et al., 2013). This protomap includes gradients of transcription factors (O’Leary et al., 2007; Cadwell et al., 2019) that vary from the rostral to caudal poles and may give rise to distinct lineages of excitatory neurons (Nowakowski et al., 2017; Bhaduri et al., 2021). Thus, it is not surprising that recent single cell transcriptomic studies found distinct expression profiles for excitatory neurons in frontal versus occipital cortical areas in mature mice (Saunders et al., 2018; Tasic et al., 2018; Bhattacherjee et al., 2019). This suggests that excitatory neuron types are unique in these regions, and thus might form area-specific local circuits that result in functional specialization. However, despite their differences, the major classes of excitatory neurons are conserved throughout the cortex (Yao et al., 2021). In agreement, we found greater differential expression of circuit-related genes between major classes within a cortical region than between best matched classes across regions. However, our analysis of class-relevant genes within each region revealed several that may lead to region-specific circuits. Thus, whether excitatory neurons form conserved circuit motifs organized by class remains an open question. An important next step is to determine which of these genes mediates local circuits versus long-range afferent and efferent connectivity. Our analysis provides several candidate genes to investigate these possibilities.

In contrast to excitatory neurons, inhibitory interneurons are produced from progenitor pools within transient embryonic structures in the ventral telencephalon (Lavdas et al., 1999; Nery et al., 2002; Butt et al., 2005; Miyoshi et al., 2007, 2010). Immature interneurons migrate to the dorsal telencephalon, where they transition to tangential migration to disperse across the entire developing cortex and hippocampus (Anderson et al., 1997; Ang et al., 2003; Marin, 2013; Mayer et al., 2015; Pelkey et al., 2017). Because interneurons throughout the mature cortex have a common embryonic origin, it is not surprising that the transcriptomic profiles of major classes are uniform from the rostral to caudal poles (Saunders et al., 2018; Tasic et al., 2018). Thus, interneurons have been assumed to form canonical circuit motifs that depend on their class, regardless of cortical or hippocampal region (Kepecs and Fishell, 2014; Pelkey et al., 2017; Huang and Paul, 2019). However, regional cues play a role in interneuron differentiation and maturation (Ishino et al., 2017; Quattrocolo et al., 2017; Petros, 2018), and relationships between major classes of excitatory and inhibitory neurons determine cortical circuit organization. For example, PT-type cells guide the radial migration of PV+ and SST+ interneurons (Lodato et al., 2011), and IT-type cells guide migration of VIP+ interneurons (Wester et al., 2019). Thus, it is possible that variations in major excitatory classes in different cortical regions result in unique inhibitory circuit motifs. Indeed, recent work using monosynaptic rabies tracing suggests both long-range afferent and local circuit connectivity of PV+ and SST+ interneurons is region dependent (Pouchelon et al., 2021). We found that expression of circuit-related genes depends mostly on major interneuron class rather than cortical region. Thus, our analysis supports the hypothesis that interneurons engage in canonical circuit motifs across the brain. However, we did identify genes that were unique for each interneuron class between ALM and VISp and may guide regional inhibitory circuit motifs.

### Directions for future studies of neocortical circuit organization

Our analysis provides clear targets for investigating cortical circuit motifs and the molecular mechanisms underlying their organization and function. Importantly, we associated genes that regulate synapse formation, maintenance, and plasticity with specific classes of excitatory and inhibitory neurons. Thus, our analysis can be used to test hypotheses regarding the function of these genes in different cell types and brain regions using Cre mouse lines that allow conditional deletion in excitatory projection neurons (Gerfen et al., 2013; Matho et al., 2021) and inhibitory interneurons (Taniguchi et al., 2011; He et al., 2016). For example, *cdh13* encodes a cadherin previously shown to be selectively expressed in PT-type excitatory neurons to guide axon targeting (Alcamo et al., 2008; Hayano et al., 2014). Importantly, our analysis identified *cdh13* as a circuit-related gene that is class-relevant to L5 PT cells in both ALM and VISp (Figure 7A). Interestingly, in the hippocampus, *cdh13* is preferentially expressed in the presynaptic terminals of SST+ dendrite-targeting interneurons, where it modulates GABA release onto excitatory pyramidal neurons (Rivero et al., 2015). In the neocortex, we also identified *cdh13* as class-relevant to SST+ interneurons (Figure 7C). In deep cortical layers, SST+ interneurons and PT-type excitatory neurons form a disynaptic feedback inhibition circuit motif (Le Be et al., 2007; Silberberg and Markram, 2007). An intriguing hypothesis is that *cdh13* plays an important role in the formation or maintenance of this motif.

We identified few genes that uniformly delineate excitatory or inhibitory classes across ALM and VISp (Figure 10). However, the genes we did find were previously shown to play important roles in the regionalization of the rostral and caudal neocortical poles. For example, *id2* is preferentially expressed in the caudal half of cortex (Rubenstein et al., 1999), and *lmo4* is preferentially expressed in motor cortex relative to visual cortex (Cederquist et al., 2013). Interestingly, our analysis also identified *tenm2* as being preferentially expressed in VISp relative to ALM (Figure 10A). Strikingly, *tenm2* knock out mice exhibit visual but not motor deficits (Young et al., 2013), which provides a functional validation of our results. Finally, we identified novel genes that may regulate cortical regionalization. These include the histone H3 variant *cenpa*, which we found is preferentially expressed in interneurons in VISp (Figure 10B). CENP-A has mostly been studied for its role in defining centromeric nucleosomes and supporting chromatin compaction in preparation for kinetochore binding and chromosomal segregation during cell division (Zhou et al., 2022). However, recent work found that CENP-A, and other components of mitosis, are repurposed in neurons to regulate circuit formation (Del Castillo et al., 2019; Zhao et al., 2019). Indeed, in *Drosophila*, CENP-A mutants show disruptions in synaptic development and neurite growth (Zhao et al., 2019). The role of CENP-A in cortical circuits is unknown, but our analysis suggests it plays a specialized role in visual cortex inhibitory circuitry.

### Implications for understanding the circuit mechanisms of autism

A challenge to understanding ASD is its broad range of impairments in social-communication, cognition, and sensorimotor functions, due in part to disruptions in circuits across the brain (Marco et al., 2011; Whyatt and Craig, 2013; Donovan and Basson, 2017; Velmeshev et al., 2019; Chowdhury et al., 2021). A prominent hypothesis is that an imbalance in the ratio of excitation to inhibition (E/I) corrupts information processing within cortical circuits and contributes to pathology (Rubenstein and Merzenich, 2003; Sohal and Rubenstein, 2019). Indeed, accumulating evidence suggests inhibitory interneurons play an important role in neurodevelopmental disorders (Goff and Goldberg, 2019, 2021; Mossner et al., 2020; Contractor et al., 2021; Nomura, 2021; Tang et al., 2021). However, the circuit mechanisms underlying ASD remain unclear. Here, we mapped candidate ASD risk genes that encode proteins necessary for synaptic connectivity and plasticity to major neuronal classes in both ALM and VISp. Our analysis implicates cell-type specific circuit components that are conserved across the cortex or are region-specific. This suggests that in some patients targeting a canonical circuit motif may be a strategy to alleviate multiple symptoms; however, in others, therapies may require region-specific interventions.

Interestingly, we identified many circuit-related ASD risk genes in IT-type excitatory neurons and VIP+ and SST+ inhibitory interneurons that were conserved between ALM and VISp. Thus, these are attractive candidates underlying impaired canonical circuit motifs. Importantly, enrichment in these cell types is consistent with recent single cell transcriptomic analysis in patients with ASD (Parikshak et al., 2013; Velmeshev et al., 2019). In humans, Velmeshev et al. (2019) highlighted *stx1a* in IT cells and *grik1* in VIP+ cells as key genes that are down or upregulated, respectively, in ASD patients. Our analysis identified these ASD risk genes as relevant to the same neuronal classes in mice. Thus, our work is consistent with findings in ASD patients and has great potential to guide translational studies.

### Limitations of the study

We inferred molecular mechanisms of synaptic connectivity between major neuronal classes based on relative levels of mRNA expression. However, this approach suffers from some limitations (discussed in detail in Sudhof (2018)). First, mRNA and protein expression levels do not always correlate. Thus, some circuit-related genes identified in our differential expression analysis may not be relevant to connectivity biases among specific neuronal classes. Second, single-cell sequencing can have a shallow read depth and fail to detect alternative splicing events. Thus, subtle yet important gene expression differences between neuronal classes are likely not represented in the data we analyzed. However, the purpose of our analysis was to provide strong candidate molecular mechanisms to be verified and investigated in future studies. Our stringent approach to assign each gene to a distinct neuronal class likely identified many that play specialized roles in circuit organization.

Our strategy filtered genes according to the GO terms “cell-cell adhesion” (CCA), “regulation of cell-cell adhesion” (RCCA), and “regulation of transsynaptic signaling” (RTSS). Most of the genes identified in our analysis were classified as RTSS, however, some were CCA and RCCA. It is important to acknowledge that CCA and RCCA are broad terms that can include genes unrelated to synaptic connectivity. However, we found that many of the genes in our analysis classified as CCA and RCCA have known synaptic functions, and included multiple cadherins, protocadherins, and neurexins (Sudhof, 2018). Furthermore, our analysis identified other CCA and RCCA genes important for synaptic connectivity, such as *cadm1*, *nlg1*, *unc5d*, and *cxcl12* (Levinson and El-Husseini, 2005; Robbins et al., 2010; Wu et al., 2016; Sudhof, 2018). Thus, despite the generality of these GO terms for cell function, they were important to include in our analysis for investigating neural circuit organization.

Finally, each major neuronal class contains unique subtypes that may form specialized microcircuit motifs (Tasic et al., 2018; Chen et al., 2019; Kim et al., 2020; Cheung et al., 2021; Que et al., 2021). In future studies, it will be interesting to use the additional metadata provided by the Allen Institute to further refine our analysis to consider these subtypes. Indeed, recent work from another group using the Allen’s data found that VIP cells in upper and lower cortical layers can be distinguished by their transcriptomic profiles, and that these subtypes are conserved between VISp and ALM (Wu et al., 2022). Thus, this is a promising approach to reveal molecular targets underlying subtype-specific circuits that may be shared or distinct across neocortical regions.

### Conclusion

Our analysis leveraged the Allen Institute’s single-cell transcriptomic datasets to reveal candidate genes underlying cell-type-specific circuits across functionally distinct cortical regions. We conclude that major neuronal classes likely establish many canonical circuit motifs that are conserved across the neocortex. However, we also identified many genes that may regulate region-specific motifs in a cell-type-specific manner. Our analysis will hopefully serve as a resource to guide further investigation of cortical circuit connectivity and the molecular mechanisms underlying its organization.

## Materials and methods

### Datasets

We downloaded the Allen’s ALM and V1 – SMART-seq (2018) datasets^2^. These include sequencing data from 25,481 neuronal and non-neuronal cells from mice ages P51, P53–P59, and P63–P91 (Tasic et al., 2018). Using the scRNA-seq toolkit Seurat (Stuart et al., 2019), we filtered for glutamatergic and GABAergic neurons from each brain region (using the Allen’s metadata) to create four separate Seurat objects: 1) ALM glutamatergic, 2) ALM GABAergic, 3) VISp glutamatergic, and 4) VISp GABAergic. To create two final objects for analyses, we merged the ALM and VISp glutamatergic objects and the ALM and VISp GABAergic objects. We followed the Seurat standard pre-processing workflow for QC, normalization, identification of highly variable features, scaling, and UMAP dimensionality reduction. We log normalized raw counts using the Seurat function NormalizeData. For each cell, this divides the raw expression count of a gene by the total expression count from all genes, multiplies by the default scale factor of 10,000, and log-transforms (natural log) the result.

### Differential expression tests

Using brain region and class metadata, we compared expression profiles of cells from each brain region (ALM or VISp) and selected major classes (L2/3 IT, L5 IT, L5 PT, and L6 CT for glutamatergic object; Pvalb, Sst, and Vip for GABAergic object) in a pairwise manner (Figures 2A,B). The number of cells compared from each class and brain region is provided in Table 1. We used the Seurat function FindMarkers with default parameters to perform the differential expression tests using log normalized expression values as inputs. FindMarkers calculates the average log fold change (avg_logFC) of each gene within a comparison. It filters out genes that have an avg_logFC of less than 0.25 before performing a Wilcoxon rank-sum test. We selected differentially expressed genes from this set that have an adjusted P-value of < 0.05 based on Bonferroni correction. All differential expression tests were conducted with classes in the same order. For example, the test between SST vs PV in ALM and VISp were both performed relative to expression in SST cells. This allowed the sign of the avg_logFC values of differentially expressed genes to be directly compared for analogous tests in ALM and VISp (e.g., a positive avg_logFC value for Gene X between SST vs PV cells in both ALM and VISp indicates Gene X is enriched in SST relative to PV in both ALM and VISp).

### Approach to normalize data presented in the Circos plots

For each pairwise comparison, we normalized the number of differentially expressed genes by the total number of genes expressed within a pair (e.g., PV vs SST cells in ALM). To determine the total within each pair, we used the AverageExpression function in Seurat to identify genes with average expression within a pair greater than 0, indicating expression in at least one member. For example, L2/3 IT and L5 IT cells in ALM express a total of 33,581 genes combined. Between them, 465 are differentially expressed for a normalized percentage of 1.4% differentially expressed genes. Percentage calculations for each test are shown in the ‘Circos Table’ of Supplementary Table 1). We organized these results in a matrix presented in the Circos plot in (Figure 2C; Krzywinski et al., 2009).

### Filtering for circuit-related genes

To identify genes that are circuit-related, we used the classification software PANTHER 16.0 (Mi et al., 2021). We selected Mus musculus as the reference organism and ran a statistical overrepresentation test (GO biological process complete) with no corrections. We selected genes labeled under the gene ontology categories “Cell-cell adhesion”, “Regulation of cell-cell adhesion”, and “Regulation of trans-synaptic signaling”.

### Labeling for clinically relevant genes

We downloaded the 2021 list of autism risk genes from the SFARI Human Gene Module (see text footnote 1) and labeled differentially expressed genes that are risk genes. Risk genes from all categories (“Suggestive Evidence,” “Strong Candidate,” “High Confidence,” and “Syndromic”) were considered.

### Strategy to determine the regional-specificity of each gene

For our analysis, we consider a differential expression datapoint as a coordinate of the following features: Comparison (Glut/GABA ALM/VISp Class 1 vs Class 2 or Glut/GABA ALM vs VISp Class 1), gene, average log fold change (avg_logFC) value, relevant GO terms, and relevant conditions (see Combined_filtered_GO.csv file for datapoints on GitHub). We created the tool DE_Collapser in Python to identify differential expression datapoints that are “ALM-specific”, “VISp-specific”, “conserved” or “divergent” (examples in Figure 4A). This was accomplished using the following algorithm. DE_Collapser takes differential expression datapoints as an input. DE_Collapser iterates through pairs of comparisons to identify those that are analogous across brain regions (e.g., GABA ALM SST vs PV is analogous to GABA VISp SST vs PV). It identifies genes that are differentially expressed in both comparisons. For each of these genes, DE_Collapser determines if the relative expression between classes is consistent across comparisons or different by using the sign of the avg_logFC. The avg_logFC values can be used because classes were compared in the same order in analogous differential expression tests (see above). Genes exhibiting a consistent relationship across regions are labeled ‘conserved’. For example, *shisa6* is enriched in VIP cells relative to PV cells in ALM and VISp (Figure 4Aiii). This is indicated by a positive avg_logFC value in the ALM and VISp comparisons. Genes exhibiting a relationship that is reversed across regions are labeled ‘divergent’. For example, L5 IT cells have greater expression of *calb1* relative to L5 PT cells in ALM but this relationship is reversed in VISp (Figure 4Aiv). This is indicated by opposite signs of the avg_logFC values for the comparison in ALM versus that in VISp. For conserved genes, DE_Collapser computes the average of the two avg_logFC values to convert a pair of differential expression datapoints into one datapoint with a single avg_logFC value. Datapoints for divergent genes are separated into a distinct subset that preserves avg_logFC values for comparisons in each region (i.e., not averaged like for Converved genes). For genes that are differentially expressed in only one of two analogous comparisons, DE_Collapser identifies if they are differentially expressed in the ALM comparison (label ‘ALM-specific’; Figure 4Ai) or VISp comparison (label ‘VISp-specific’; Figure 4Aii). Examples of applying DE_Collapser on raw datapoints is represented by the set of arrows between Figure 5A and Figure 5B.

### Strategy to identify class-relevant genes

All the following class analyses presented in Figures 5-10 were performed separately for glutamatergic and GABAergic data. To determine if differentially expressed genes map to a class, we created Subclass Identifier (SCID). This algorithm takes the output of DE_Collapser that includes conserved or region-specific data (not divergent data). It separates comparisons into the order of their constituent classes. For example, in a VIP vs PV comparison, VIP is the first class and PV is the second class. Based on the avg_logFC values, SCID identifies which class has greater or lesser relative expression of each gene: If the avg_logFC > 0 for gene A in a VIP vs PV comparison, then the first class (VIP) has greater expression and second class (PV) has lesser expression. If the avg_logFC < 0 for gene B in a VIP vs PV comparison, then the second class (PV) has greater expression and first class (VIP) has lesser expression.

To identify class-relevant genes, the results from differential expression tests were required to be consistent for the following comparisons, which we call class sets:

L2/3 IT = {’L2/3 IT vs. L5 IT’, ‘L2/3 IT vs. L6 CT’, ‘L2/3 IT vs. L5 PT’}
L5 IT = {’L2/3 IT vs. L5 IT’,’L5 IT vs. L5 PT’,’L5 IT vs. L6 CT’}
IT = {’L2/3 IT vs. L5 PT’, ‘L2/3 IT vs. L6 CT’, ‘L5 IT vs. L5 PT’,’L5 IT vs. L6 CT’}
L5 PT = {’L2/3 IT vs. L5 PT’,’L5 IT vs. L5 PT’,’L5 PT vs. L6 CT’}
L6 CT = {’L2/3 IT vs. L6 CT’,’L5 IT vs. L6 CT’,’L5 PT vs. L6 CT’}
VIP = {’VIP vs. PV’, ‘VIP vs. SST’}
SST = {’SST vs. PV’, ‘VIP vs. SST’}
PV = {’SST vs. PV’, ‘VIP vs. PV’}

The following steps comprise the key computations of the algorithm, which we demonstrate with an example considering a hypothetical gene X. We refer to a comparison set as all the comparisons that a gene appears differentially expressed in.

(1) SCID iterates through the differential expression data for each gene and determines the list of classes that differentially express the gene. If L2/3 IT and L5 IT are identified, IT is added to the class list.
  • Example: If gene X is differentially expressed in the comparison set {‘VIP vs PV’, ‘VIP vs SST’, ‘SST vs PV’}, then SCID identifies SST, VIP, and PV as the class list.
(2) For each class in the class list, SCID iterates through pertinent class sets that we define above. It identifies class sets that are a subset of the comparison set associated with the gene.
  • Example: For gene X, the VIP, {’VIP vs PV’, ‘VIP vs SST’}; SST, {’SST vs PV’, ‘VIP vs SST’}; and PV {’SST vs PV’, ‘VIP vs PV’} class sets would each be identified as a subset of the comparison set {’VIP vs PV’, ‘VIP vs SST’, ‘SST vs PV’}.
(3) For each class set, SCID filters the differential expression data for comparisons that are included in the set.
  • Example: For the VIP set, SCID filters for gene X data involving ‘VIP vs PV’, ‘VIP vs SST’ comparisons; for the SST set, it filters for gene X data involving ‘SST vs PV’, ‘VIP vs SST’ comparisons; and for the PV set, it filters for gene X data involving ‘SST vs PV’, ‘VIP vs PV’ comparisons.
(4) In each filtered dataset, SCID evaluates which class has greater or lesser expression of a gene in each comparison. This enables it to determine if the class (e.g., VIP) corresponding to the class set (e.g. {VIP vs. PV’, ‘VIP vs. SST’}) has consistent expression relative to all other classes (e.g., PV and SST). We refer to these as ‘class-relevant’ genes. We label class-relevant genes for their class and the corresponding expression relative to all other classes (Figures 5C, 7-9). Genes that are not class-relevant remain unlabeled.
  • Example: If gene X is expressed greater in VIP cells relative to PV and SST cells, and expressed less in SST cells relative to VIP and PV cells, gene X is labeled as up in VIP cells and down in SST cells.

Note: Given the low number of genes in the divergent subset, we searched for class-relevant genes directly. No genes were identified. Also, per the defined class lists and differential expression analysis, SCID identified features are relative to either glutamatergic or GABAergic classes.

### Strategy to identify class-relevant genes that involve conserved and region-specific data

For the genes that require different class-relevant labels in the conserved and region-specific sets (Figure 6), we created CortexSCID. This algorithm compares the results of performing SCID on (1) the conserved data and the raw ALM data or (2) the conserved data and the raw VISp data. CortexSCID identifies genes that are present in the conserved set and ALM or VISp sets and removes genes that have the same class labels. The remaining genes are relevant to one or more classes only in ALM or VISp. We refer to these as * genes if the same gene is relevant to different classes in a conserved and region-specific manner (e.g., egr1 in Figure 6). CortexSCID identifies * genes, their associated classes, and relative expression for each class. It updates the ALM or VISp differential expression data by preserving only genes and their class-relevance that are region-specific.

### Identifying genes that are biased to ALM or VISp

Using the differential expression data for comparisons between the same class across ALM and VISp (Figure 2B), we queried for genes that are enriched across multiple glutamatergic or GABAergic classes in ALM relative to VISp or vice versa (Figure 10). For example, we identified *cenpa* as being enriched in VIP, PV, and SST cells in VISp relative to their equivalents in ALM (Figure 10B left, Figure 10D). We also searched for genes that are differentially expressed by VIP cells but not PV or SST cells across brain regions (Figure 10B right, Figure 10E) or PV and SST cells but not VIP cells across brain regions (no such genes identified). The Violin plots for *tenm2*, *cenpa*, and *igf1* were created in Seurat (Figures 10C–E).

### Heatmap creation

We created the function Morpheus Prepper (MorphPrep) to streamline class and clinical analyses and organize results as matrices for heatmap visualization in Morpheus^3^.

## Data availability statement

Publicly available datasets were analyzed in this study. These data can be found here: https://portal.brain-map.org/atlases-and-data/rnaseq. We uploaded our annotated code and output data to GitHub. We include a README file that provides instructions for performing analyses and visualizing the results, and an overview of each data file. These files and data can be found here: https://github.com/Moussa-A/Wester-Lab.

## Author contributions

AM and JW designed the study, analyzed the data, and wrote the manuscript. AM wrote custom data analysis routines in Python. Both authors contributed to the article and approved the submitted version.

## Footnotes

1
https://gene.sfari.org/database/human-gene/

2
https://portal.brain-map.org/atlases-and-data/rnaseq

3
https://software.broadinstitute.org/morpheus

## Supplementary material

The Supplementary Material for this article can befound online at: https://www.frontiersin.org/articles/10.3389/fncir.2022.982721/full#supplementary-material

## References

[B1] Alcamo E. A. Chirivella L. Dautzenberg M. Dobreva G. Farinas I. Grosschedl R. (2008). Satb2 regulates callosal projection neuron identity in the developing cerebral cortex. *Neuron* 57 364–377. 10.1016/j.neuron.2007.12.012 18255030

[B2] Anderson S. A. Eisenstat D. D. Shi L. Rubenstein J. L. (1997). Interneuron migration from basal forebrain to neocortex: Dependence on Dlx genes. *Science* 278 474–476. 10.1126/science.278.5337.474 9334308

[B3] Ang E. S. Jr. Haydar T. F. Gluncic V. Rakic P. (2003). Four-dimensional migratory coordinates of GABAergic interneurons in the developing mouse cortex. *J. Neurosci.* 23 5805–5815. 10.1523/JNEUROSCI.23-13-05805.2003 12843285 PMC6741259

[B4] Bhaduri A. Sandoval-Espinosa C. Otero-Garcia M. Oh I. Yin R. Eze U. C. (2021). An atlas of cortical arealization identifies dynamic molecular signatures. *Nature* 598 200–204. 10.1038/s41586-021-03910-8 34616070 PMC8494648

[B5] Bhattacherjee A. Djekidel M. N. Chen R. Chen W. Tuesta L. M. Zhang Y. (2019). Cell type-specific transcriptional programs in mouse prefrontal cortex during adolescence and addiction. *Nat. Commun.* 10:4169. 10.1038/s41467-019-12054-3 31519873 PMC6744514

[B6] Brown S. P. Hestrin S. (2009b). Intracortical circuits of pyramidal neurons reflect their long-range axonal targets. *Nature* 457 1133–1136. 10.1038/nature07658 19151698 PMC2727746

[B7] Brown S. P. Hestrin S. (2009a). Cell-type identity: A key to unlocking the function of neocortical circuits. *Curr. Opin. Neurobiol.* 19 415–421. 10.1016/j.conb.2009.07.011 19674891 PMC2739254

[B8] Brumback A. C. Ellwood I. T. Kjaerby C. Iafrati J. Robinson S. Lee A. T. (2018). Identifying specific prefrontal neurons that contribute to autism-associated abnormalities in physiology and social behavior. *Mol. Psychiatry* 23 2078–2089. 10.1038/mp.2017.213 29112191 PMC6594833

[B9] Butt S. J. Fuccillo M. Nery S. Noctor S. Kriegstein A. Corbin J. G. (2005). The temporal and spatial origins of cortical interneurons predict their physiological subtype. *Neuron* 48 591–604. 10.1016/j.neuron.2005.09.034 16301176

[B10] Cadwell C. R. Bhaduri A. Mostajo-Radji M. A. Keefe M. G. Nowakowski T. J. (2019). Development and Arealization of the Cerebral Cortex. *Neuron* 103 980–1004. 10.1016/j.neuron.2019.07.009 31557462 PMC9245854

[B11] Cederquist G. Y. Azim E. Shnider S. J. Padmanabhan H. Macklis J. D. (2013). Lmo4 establishes rostral motor cortex projection neuron subtype diversity. *J. Neurosci.* 33 6321–6332. 10.1523/JNEUROSCI.5140-12.2013 23575831 PMC3698850

[B12] Chen X. Sun Y. C. Zhan H. Kebschull J. M. Fischer S. Matho K. (2019). High-Throughput Mapping of Long-Range Neuronal Projection Using In Situ Sequencing. *Cell* 179:772–786.e719. 10.1016/j.cell.2019.09.023 31626774 PMC7836778

[B13] Cheung V. Chung P. Bjorni M. Shvareva V. A. Lopez Y. C. Feinberg E. H. (2021). Virally encoded connectivity transgenic overlay RNA sequencing (VECTORseq) defines projection neurons involved in sensorimotor integration. *Cell Rep.* 37:110131. 10.1016/j.celrep.2021.110131 34936877 PMC8719358

[B14] Chowdhury D. Watters K. Biederer T. (2021). Synaptic recognition molecules in development and disease. *Curr. Top. Dev. Biol.* 142 319–370. 10.1016/bs.ctdb.2020.12.009 33706921 PMC8632550

[B15] Contractor A. Ethell I. M. Portera-Cailliau C. (2021). Cortical interneurons in autism. *Nat. Neurosci.* 24 1648–1659. 10.1038/s41593-021-00967-6 34848882 PMC9798607

[B16] Del Castillo U. Norkett R. Gelfand V. I. (2019). Unconventional Roles of Cytoskeletal Mitotic Machinery in Neurodevelopment. *Trends Cell Biol.* 29 901–911. 10.1016/j.tcb.2019.08.006 31597609 PMC6827560

[B17] Del Rosario J. Speed A. Arrowood H. Motz C. Pardue M. Haider B. (2021). Diminished Cortical Excitation and Elevated Inhibition During Perceptual Impairments in a Mouse Model of Autism. *Cereb. Cortex* 31 3462–3474. 10.1093/cercor/bhab025 33677512 PMC8525192

[B18] Donovan A. P. Basson M. A. (2017). The neuroanatomy of autism - a developmental perspective. *J. Anat.* 230 4–15. 10.1111/joa.12542 27620360 PMC5192959

[B19] Douglas R. J. Martin K. A. (2004). Neuronal circuits of the neocortex. *Annu. Rev. Neurosci.* 27 419–451. 10.1146/annurev.neuro.27.070203.144152 15217339

[B20] Elsen G. E. Hodge R. D. Bedogni F. Daza R. A. Nelson B. R. Shiba N. (2013). The protomap is propagated to cortical plate neurons through an Eomes-dependent intermediate map. *Proc. Natl. Acad. Sci. U.S.A.* 110 4081–4086. 10.1073/pnas.1209076110 23431145 PMC3593833

[B21] Földy C. Darmanis S. Aoto J. Malenka R. C. Quake S. R. Südhof T. C. (2016). Single-cell RNAseq reveals cell adhesion molecule profiles in electrophysiologically defined neurons. *Proc. Natl. Acad. Sci. U.S.A.* 113:E5222–E5231. 10.1073/pnas.1610155113 27531958 PMC5024636

[B22] Fuccillo M. V. Földy C. Gökce Ö Rothwell P. E. Sun G. L. Malenka R. C. (2015). Single-Cell mRNA Profiling Reveals Cell-Type-Specific Expression of Neurexin Isoforms. *Neuron* 87 326–340. 10.1016/j.neuron.2015.06.028 26182417 PMC4733560

[B23] Gerfen C. R. Paletzki R. Heintz N. (2013). GENSAT BAC cre-recombinase driver lines to study the functional organization of cerebral cortical and basal ganglia circuits. *Neuron* 80 1368–1383. 10.1016/j.neuron.2013.10.016 24360541 PMC3872013

[B24] Goff K. M. Goldberg E. M. (2019). Vasoactive intestinal peptide-expressing interneurons are impaired in a mouse model of Dravet syndrome. *eLife* 8:e46846. 10.7554/eLife.46846 31282864 PMC6629374

[B25] Goff K. M. Goldberg E. M. (2021). A Role for Vasoactive Intestinal Peptide Interneurons in Neurodevelopmental Disorders. *Dev. Neurosci.* 43 168–180. 10.1159/000515264 33794534 PMC8440337

[B26] Greig L. C. Woodworth M. B. Galazo M. J. Padmanabhan H. Macklis J. D. (2013). Molecular logic of neocortical projection neuron specification, development and diversity. *Nat. Rev. Neurosci.* 14 755–769. 10.1038/nrn3586 24105342 PMC3876965

[B27] Gutman-Wei A. Y. Brown S. P. (2021). Mechanisms Underlying Target Selectivity for Cell Types and Subcellular Domains in Developing Neocortical Circuits. *Front. Neural. Circuits* 15:728832. 10.3389/fncir.2021.728832 34630048 PMC8497978

[B28] Harris K. D. Shepherd G. M. (2015). The neocortical circuit: Themes and variations. *Nat. Neurosci.* 18 170–181. 10.1038/nn.3917 25622573 PMC4889215

[B29] Hayano Y. Zhao H. Kobayashi H. Takeuchi K. Norioka S. Yamamoto N. (2014). The role of T-cadherin in axonal pathway formation in neocortical circuits. *Development* 141 4784–4793. 10.1242/dev.108290 25468941

[B30] He M. Tucciarone J. Lee S. Nigro M. J. Kim Y. Levine J. M. (2016). Strategies and Tools for Combinatorial Targeting of GABAergic Neurons in Mouse Cerebral Cortex. *Neuron* 91 1228–1243. 10.1016/j.neuron.2016.08.021 27618674 PMC5223593

[B31] Huang Z. J. Paul A. (2019). The diversity of GABAergic neurons and neural communication elements. *Nat. Rev. Neurosci.* 20 563–572. 10.1038/s41583-019-0195-4 31222186 PMC8796706

[B32] Ishino Y. Yetman M. J. Sossi S. M. Steinecke A. Hayano Y. Taniguchi H. (2017). Regional Cellular Environment Shapes Phenotypic Variations of Hippocampal and Neocortical Chandelier Cells. *J. Neurosci.* 37 9901–9916. 10.1523/JNEUROSCI.0047-17.2017 28912162 PMC6596599

[B33] Karnani M. M. Jackson J. Ayzenshtat I. Hamzehei Sichani A. Manoocheri K. Kim S. (2016). Opening Holes in the Blanket of Inhibition: Localized Lateral Disinhibition by VIP Interneurons. *J. Neurosci.* 36 3471–3480. 10.1523/JNEUROSCI.3646-15.2016 27013676 PMC4804006

[B34] Kepecs A. Fishell G. (2014). Interneuron cell types are fit to function. *Nature* 505 318–326. 10.1038/nature12983 24429630 PMC4349583

[B35] Kim E. J. Zhang Z. Huang L. Ito-Cole T. Jacobs M. W. Juavinett A. L. (2020). Extraction of Distinct Neuronal Cell Types from within a Genetically Continuous Population. *Neuron* 107:274–282.e276. 10.1016/j.neuron.2020.04.018 32396852 PMC7381365

[B36] Kiritani T. Wickersham I. R. Seung H. S. Shepherd G. M. (2012). Hierarchical connectivity and connection-specific dynamics in the corticospinal-corticostriatal microcircuit in mouse motor cortex. *J. Neurosci.* 32 4992–5001. 10.1523/JNEUROSCI.4759-11.2012 22492054 PMC3329752

[B37] Krzywinski M. Schein J. Birol I. Connors J. Gascoyne R. Horsman D. (2009). Circos: An information aesthetic for comparative genomics. *Genome Res.* 19 1639–1645. 10.1101/gr.092759.109 19541911 PMC2752132

[B38] Kubota Y. (2014). Untangling GABAergic wiring in the cortical microcircuit. *Curr. Opin. Neurobiol.* 26 7–14. 10.1016/j.conb.2013.10.003 24650498

[B39] Lavdas A. A. Grigoriou M. Pachnis V. Parnavelas J. G. (1999). The medial ganglionic eminence gives rise to a population of early neurons in the developing cerebral cortex. *J. Neurosci.* 19 7881–7888. 10.1523/JNEUROSCI.19-18-07881.1999 10479690 PMC6782477

[B40] Le Be J. V. Silberberg G. Wang Y. Markram H. (2007). Morphological, electrophysiological, and synaptic properties of corticocallosal pyramidal cells in the neonatal rat neocortex. *Cereb. Cortex* 17 2204–2213. 10.1093/cercor/bhl127 17124287

[B41] Lee A. T. Gee S. M. Vogt D. Patel T. Rubenstein J. L. Sohal V. S. (2014). Pyramidal neurons in prefrontal cortex receive subtype-specific forms of excitation and inhibition. *Neuron* 81 61–68. 10.1016/j.neuron.2013.10.031 24361076 PMC3947199

[B42] Lee S. Hjerling-Leffler J. Zagha E. Fishell G. Rudy B. (2010). The largest group of superficial neocortical GABAergic interneurons expresses ionotropic serotonin receptors. *J. Neurosci.* 30 16796–16808. 10.1523/JNEUROSCI.1869-10.2010 21159951 PMC3025500

[B43] Lee S. Kruglikov I. Huang Z. J. Fishell G. Rudy B. (2013). A disinhibitory circuit mediates motor integration in the somatosensory cortex. *Nat. Neurosci.* 16 1662–1670. 10.1038/nn.3544 24097044 PMC4100076

[B44] Levinson J. N. El-Husseini A. (2005). Building excitatory and inhibitory synapses: Balancing neuroligin partnerships. *Neuron* 48 171–174. 10.1016/j.neuron.2005.09.017 16242398

[B45] Lodato S. Rouaux C. Quast K. B. Jantrachotechatchawan C. Studer M. Hensch T. K. (2011). Excitatory projection neuron subtypes control the distribution of local inhibitory interneurons in the cerebral cortex. *Neuron* 69 763–779. 10.1016/j.neuron.2011.01.015 21338885 PMC3061282

[B46] Luo L. (2021). Architectures of neuronal circuits. *Science* 373:eabg7285. 10.1126/science.abg7285 34516844 PMC8916593

[B47] Marco E. J. Hinkley L. B. Hill S. S. Nagarajan S. S. (2011). Sensory processing in autism: A review of neurophysiologic findings. *Pediatr. Res.* 69:48r–54r. 10.1203/PDR.0b013e3182130c54 21289533 PMC3086654

[B48] Mardinly A. R. Spiegel I. Patrizi A. Centofante E. Bazinet J. E. Tzeng C. P. (2016). Sensory experience regulates cortical inhibition by inducing IGF1 in VIP neurons. *Nature* 531 371–375. 10.1038/nature17187 26958833 PMC4823817

[B49] Marin O. (2013). Cellular and molecular mechanisms controlling the migration of neocortical interneurons. *Eur. J. Neurosci.* 38 2019–2029. 10.1111/ejn.12225 23651101

[B50] Matho K. S. Huilgol D. Galbavy W. He M. Kim G. An X. (2021). Genetic dissection of the glutamatergic neuron system in cerebral cortex. *Nature* 598 182–187. 10.1038/s41586-021-03955-9 34616069 PMC8494647

[B51] Mayer C. Jaglin X. H. Cobbs L. V. Bandler R. C. Streicher C. Cepko C. L. (2015). Clonally Related Forebrain Interneurons Disperse Broadly across Both Functional Areas and Structural Boundaries. *Neuron* 87 989–998. 10.1016/j.neuron.2015.07.011 26299473 PMC4560602

[B52] Mi H. Ebert D. Muruganujan A. Mills C. Albou L. P. Mushayamaha T. (2021). PANTHER version 16: A revised family classification, tree-based classification tool, enhancer regions and extensive API. *Nucleic Acids Res.* 49:D394–D403. 10.1093/nar/gkaa1106 33290554 PMC7778891

[B53] Miyoshi G. Butt S. J. Takebayashi H. Fishell G. (2007). Physiologically distinct temporal cohorts of cortical interneurons arise from telencephalic Olig2-expressing precursors. *J. Neurosci.* 27 7786–7798. 10.1523/JNEUROSCI.1807-07.2007 17634372 PMC6672881

[B54] Miyoshi G. Hjerling-Leffler J. Karayannis T. Sousa V. H. Butt S. J. Battiste J. (2010). Genetic fate mapping reveals that the caudal ganglionic eminence produces a large and diverse population of superficial cortical interneurons. *J. Neurosci.* 30 1582–1594. 10.1523/JNEUROSCI.4515-09.2010 20130169 PMC2826846

[B55] Morishima M. Kawaguchi Y. (2006). Recurrent connection patterns of corticostriatal pyramidal cells in frontal cortex. *J. Neurosci.* 26 4394–4405. 10.1523/JNEUROSCI.0252-06.2006 16624959 PMC6674016

[B56] Mossner J. M. Batista-Brito R. Pant R. Cardin J. A. (2020). Developmental loss of MeCP2 from VIP interneurons impairs cortical function and behavior. *eLife* 9:e55639. 10.7554/eLife.55639 32343226 PMC7213975

[B57] Nery S. Fishell G. Corbin J. G. (2002). The caudal ganglionic eminence is a source of distinct cortical and subcortical cell populations. *Nat. Neurosci.* 5 1279–1287. 10.1038/nn971 12411960

[B58] Nomura T. (2021). Interneuron Dysfunction and Inhibitory Deficits in Autism and Fragile X Syndrome. *Cells* 10:2610. 10.3390/cells10102610 34685590 PMC8534049

[B59] Nowakowski T. J. Bhaduri A. Pollen A. A. Alvarado B. Mostajo-Radji M. A. Di Lullo E. (2017). Spatiotemporal gene expression trajectories reveal developmental hierarchies of the human cortex. *Science* 358 1318–1323. 10.1126/science.aap8809 29217575 PMC5991609

[B60] O’Leary D. D. Chou S. J. Sahara S. (2007). Area patterning of the mammalian cortex. *Neuron* 56 252–269. 10.1016/j.neuron.2007.10.010 17964244

[B61] Parikshak N. N. Luo R. Zhang A. Won H. Lowe J. K. Chandran V. (2013). Integrative functional genomic analyses implicate specific molecular pathways and circuits in autism. *Cell* 155 1008–1021. 10.1016/j.cell.2013.10.031 24267887 PMC3934107

[B62] Paul A. Crow M. Raudales R. He M. Gillis J. Huang Z. J. (2017). Transcriptional Architecture of Synaptic Communication Delineates GABAergic Neuron Identity. *Cell* 171:522–539e520. 10.1016/j.cell.2017.08.032 28942923 PMC5772785

[B63] Pelkey K. A. Chittajallu R. Craig M. T. Tricoire L. Wester J. C. Mcbain C. J. (2017). Hippocampal GABAergic Inhibitory Interneurons. *Physiol. Rev.* 97 1619–1747. 10.1152/physrev.00007.2017 28954853 PMC6151493

[B64] Petros T. J. (2018). Stranger in a Strange Land: Using Heterotopic Transplantations to Study Nature vs Nurture in Brain Development. *J. Exp. Neurosci.* 12:1179069518758656. 10.1177/1179069518758656 29511360 PMC5833213

[B65] Pfeffer C. K. Xue M. He M. Huang Z. J. Scanziani M. (2013). Inhibition of inhibition in visual cortex: The logic of connections between molecularly distinct interneurons. *Nat. Neurosci.* 16 1068–1076. 10.1038/nn.3446 23817549 PMC3729586

[B66] Pi H. J. Hangya B. Kvitsiani D. Sanders J. I. Huang Z. J. Kepecs A. (2013). Cortical interneurons that specialize in disinhibitory control. *Nature* 503 521–524. 10.1038/nature12676 24097352 PMC4017628

[B67] Pouchelon G. Dwivedi D. Bollmann Y. Agba C. K. Xu Q. Mirow A. M. C. (2021). The organization and development of cortical interneuron presynaptic circuits are area specific. *Cell Rep.* 37:109993. 10.1016/j.celrep.2021.109993 34758329 PMC8832360

[B68] Quattrocolo G. Fishell G. Petros T. J. (2017). Heterotopic Transplantations Reveal Environmental Influences on Interneuron Diversity and Maturation. *Cell Rep.* 21 721–731. 10.1016/j.celrep.2017.09.075 29045839 PMC5662128

[B69] Que L. Lukacsovich D. Luo W. Földy C. (2021). Transcriptional and morphological profiling of parvalbumin interneuron subpopulations in the mouse hippocampus. *Nat. Commun.* 12:108. 10.1038/s41467-020-20328-4 33398060 PMC7782706

[B70] Rakic P. (1988). Specification of cerebral cortical areas. *Science* 241 170–176. 10.1126/science.3291116 3291116

[B71] Rivero O. Selten M. M. Sich S. Popp S. Bacmeister L. Amendola E. (2015). Cadherin-13, a risk gene for ADHD and comorbid disorders, impacts GABAergic function in hippocampus and cognition. *Transl. Psychiatry* 5:e655. 10.1038/tp.2015.152 26460479 PMC4930129

[B72] Robbins E. M. Krupp A. J. Perez De Arce K. Ghosh A. K. Fogel A. I. Boucard A. (2010). SynCAM 1 adhesion dynamically regulates synapse number and impacts plasticity and learning. *Neuron* 68 894–906. 10.1016/j.neuron.2010.11.003 21145003 PMC3026433

[B73] Rubenstein J. L. Anderson S. Shi L. Miyashita-Lin E. Bulfone A. Hevner R. (1999). Genetic control of cortical regionalization and connectivity. *Cereb. Cortex* 9 524–532. 10.1093/cercor/9.6.524 10498270

[B74] Rubenstein J. L. Merzenich M. M. (2003). Model of autism: Increased ratio of excitation/inhibition in key neural systems. *Genes Brain Behav.* 2 255–267. 10.1034/j.1601-183X.2003.00037.x 14606691 PMC6748642

[B75] Saunders A. Macosko E. Z. Wysoker A. Goldman M. Krienen F. M. De Rivera H. (2018). Molecular Diversity and Specializations among the Cells of the Adult Mouse Brain. *Cell* 174:1015–1030 e1016. 10.1016/j.cell.2018.07.028 30096299 PMC6447408

[B76] Silberberg G. Markram H. (2007). Disynaptic inhibition between neocortical pyramidal cells mediated by Martinotti cells. *Neuron* 53 735–746. 10.1016/j.neuron.2007.02.012 17329212

[B77] Smith A. L. Jung E. M. Jeon B. T. Kim W. Y. (2020). Arid1b haploinsufficiency in parvalbumin- or somatostatin-expressing interneurons leads to distinct ASD-like and ID-like behavior. *Sci. Rep.* 10:7834. 10.1038/s41598-020-64066-5 32398858 PMC7217886

[B78] Sohal V. S. Rubenstein J. L. R. (2019). Excitation-inhibition balance as a framework for investigating mechanisms in neuropsychiatric disorders. *Mol. Psychiatry* 24 1248–1257. 10.1038/s41380-019-0426-0 31089192 PMC6742424

[B79] Stuart T. Butler A. Hoffman P. Hafemeister C. Papalexi E. Mauck W. M. III (2019). Comprehensive Integration of Single-Cell Data. *Cell* 177:1888–1902.e1821. 10.1016/j.cell.2019.05.031 31178118 PMC6687398

[B80] Sudhof T. C. (2018). Towards an Understanding of Synapse Formation. *Neuron* 100 276–293. 10.1016/j.neuron.2018.09.040 30359597 PMC6226307

[B81] Tang X. Jaenisch R. Sur M. (2021). The role of GABAergic signalling in neurodevelopmental disorders. *Nat. Rev. Neurosci.* 22 290–307. 10.1038/s41583-021-00443-x 33772226 PMC9001156

[B82] Taniguchi H. He M. Wu P. Kim S. Paik R. Sugino K. (2011). A resource of cre driver lines for genetic targeting of GABAergic neurons in cerebral cortex. *Neuron* 71 995–1013. 10.1016/j.neuron.2011.07.026 21943598 PMC3779648

[B83] Tasic B. Menon V. Nguyen T. N. Kim T. K. Jarsky T. Yao Z. (2016). Adult mouse cortical cell taxonomy revealed by single cell transcriptomics. *Nat. Neurosci.* 19 335–346. 10.1038/nn.4216 26727548 PMC4985242

[B84] Tasic B. Yao Z. Graybuck L. T. Smith K. A. Nguyen T. N. Bertagnolli D. (2018). Shared and distinct transcriptomic cell types across neocortical areas. *Nature* 563 72–78. 10.1038/s41586-018-0654-5 30382198 PMC6456269

[B85] Tremblay R. Lee S. Rudy B. (2016). GABAergic Interneurons in the Neocortex: From Cellular Properties to Circuits. *Neuron* 91 260–292. 10.1016/j.neuron.2016.06.033 27477017 PMC4980915

[B86] Velmeshev D. Schirmer L. Jung D. Haeussler M. Perez Y. Mayer S. (2019). Single-cell genomics identifies cell type-specific molecular changes in autism. *Science* 364 685–689. 10.1126/science.aav8130 31097668 PMC7678724

[B87] Wester J. C. Mahadevan V. Rhodes C. T. Calvigioni D. Venkatesh S. Maric D. (2019). Neocortical Projection Neurons Instruct Inhibitory Interneuron Circuit Development in a Lineage-Dependent Manner. *Neuron* 102:960–975 e966. 10.1016/j.neuron.2019.03.036 31027966 PMC8965597

[B88] Whyatt C. Craig C. (2013). Sensory-motor problems in Autism. *Front. Integr. Neurosci.* 7:51. 10.3389/fnint.2013.00051 23882194 PMC3714545

[B89] Wu J. Zhao Z. Shi Y. He M. (2022). Cortical VIP(+) Interneurons in the Upper and Deeper Layers Are Transcriptionally Distinct. *J. Mol. Neurosci.* 72 1779–1795. 10.1007/s12031-022-02040-8 35708842

[B90] Wu P. R. Cho K. K. Vogt D. Sohal V. S. Rubenstein J. L. (2016). The Cytokine CXCL12 Promotes Basket Interneuron Inhibitory Synapses in the Medial Prefrontal Cortex. *Cereb. Cortex* 27 4303–4313. 10.1093/cercor/bhw230 27497284 PMC6410508

[B91] Xu Q. Cobos I. De La Cruz E. Rubenstein J. L. Anderson S. A. (2004). Origins of cortical interneuron subtypes. *J. Neurosci.* 24 2612–2622. 10.1523/JNEUROSCI.5667-03.2004 15028753 PMC6729522

[B92] Yao Z. Van Velthoven C. T. J. Nguyen T. N. Goldy J. Sedeno-Cortes A. E. Baftizadeh F. (2021). A taxonomy of transcriptomic cell types across the isocortex and hippocampal formation. *Cell* 184:3222–3241.e3226. 10.1016/j.cell.2021.04.021 34004146 PMC8195859

[B93] Ye L. Allen W. E. Thompson K. R. Tian Q. Hsueh B. Ramakrishnan C. (2016). Wiring and Molecular Features of Prefrontal Ensembles Representing Distinct Experiences. *Cell* 165 1776–1788. 10.1016/j.cell.2016.05.010 27238022 PMC5708551

[B94] Yizhar O. Fenno L. E. Prigge M. Schneider F. Davidson T. J. O’shea D. J. (2011). Neocortical excitation/inhibition balance in information processing and social dysfunction. *Nature* 477 171–178. 10.1038/nature10360 21796121 PMC4155501

[B95] Young T. R. Bourke M. Zhou X. Oohashi T. Sawatari A. Fässler R. (2013). Ten-m2 is required for the generation of binocular visual circuits. *J. Neurosci.* 33 12490–12509. 10.1523/JNEUROSCI.4708-12.2013 23884953 PMC6618674

[B96] Zeisel A. Munoz-Manchado A. B. Codeluppi S. Lonnerberg P. La Manno G. Jureus A. (2015). Brain structure. Cell types in the mouse cortex and hippocampus revealed by single-cell RNA-seq. *Science* 347 1138–1142. 10.1126/science.aaa1934 25700174

[B97] Zhao G. Oztan A. Ye Y. Schwarz T. L. (2019). Kinetochore Proteins Have a Post-Mitotic Function in Neurodevelopment. *Dev. Cell* 48:873–882.e874. 10.1016/j.devcel.2019.02.003 30827899 PMC7375515

[B98] Zhou K. Gebala M. Woods D. Sundararajan K. Edwards G. Krzizike D. (2022). CENP-N promotes the compaction of centromeric chromatin. *Nat. Struct. Mol. Biol.* 29 403–413. 10.1038/s41594-022-00758-y 35422519 PMC9010303

